# Alternative Stable States Generated by Ontogenetic Niche Shift in the Presence of Multiple Resource Use

**DOI:** 10.1371/journal.pone.0014667

**Published:** 2011-02-08

**Authors:** Takefumi Nakazawa

**Affiliations:** 1 Center for Ecological Research, Kyoto University, Otsu, Japan; 2 Institute of Oceanography, National Taiwan University, Taipei, Taiwan; University of Pretoria, South Africa

## Abstract

It has been suggested that when juveniles and adults use different resources or habitats, alternative stable states (ASS) may exist in systems coupled by an ontogenetic niche shift. However, mainly the simplest system, i.e., the one-consumer–two-resource system, has been studied previously, and little is known about the development of ASS existing in more complex systems. Here, I theoretically investigated the development of ASS caused by an ontogenetic niche shift in the presence of multiple resource use. I considered three independent scenarios; (i) additional resources, (ii) multiple habitats, and (iii) interstage resource sharing. The model analyses illustrate that relative balance between the total resource availability in the juvenile and adult habitats is crucial for the development of ASS. This balance is determined by factors such as local habitat productivity, subsidy inputs, colonization area, and foraging mobility. Furthermore, it is also shown that interstage resource sharing generally suppresses ASS. These results suggest that the anthropogenic impacts of habitat modifications (e.g., fragmentation and destruction) or interaction modifications (e.g., changes in ontogeny and foraging behavior) propagate through space and may cause or prevent regime shifts in the regional community structure.

## Introduction

Many animals change their resource or habitat use during the course of individual growth; such a change is known as ontogenetic niche shift [1], [2]. A key aspect of ontogenetic niche shift is that it divides a population into distinct life-history stages that have different trophic effects on food webs. Therefore, when an animal uses different habitats at different stages, ontogenetic niche shifts have spatially spreading demographic impacts (e.g., [3]-[6]). In the field of spatial ecology, different theories have recently been developed from a variety of viewpoints, including metacommunity (e.g., [7]), spatial subsidy and cross-ecosystem linkage (e.g., [8]), food-web theory (e.g., [9]), and meta-ecosystem (e.g., [10]). However, these theories have rarely considered ontogenetic niche shifts as a major coupling factor of spatially distinct food webs. At present, therefore, little is known about how spatial food-web dynamics are mediated by ontogenetic niches shifts, despite that such mediation is a fairly common occurrence in nature [3]-[6] (see also a review in [11]).

The ecological consequences of ontogenetic food-web coupling have been investigated in only a few recent theoretical studies [6], [11]-[13]. Notably, previous models have suggested that when juveniles and adults use different resources within their habitats, the systems coupled by ontogenetic niche shift may exhibit alternative stable states (ASS) [11]-[13]. The mechanism of ASS involves positive feedback caused by apparent competition-like interactions between juvenile and adult resources. Suppose the amount of the juvenile resource increases. This will promote maturation, and thus, negatively affect the adult resource, which in turn, leads to an increase in the juvenile resource through a suppression of reproduction. This process results in positive feedback that leads to a situation in which the system converges to either a juvenile- or an adult-dominated state, depending on the initial conditions (see also a review by [14] for details on density-dependent population regulation in stage-structured models). The existence of ASS has important implications, particularly for ecosystem management, because it suggests that sudden and abrupt shifts in a regional community structure may occur after local environmental changes have occurred in one habitat (for details on regime shifts, see [15]-[17]).

Previous theoretical studies have only considered one consumer-two resource systems [6], [12], [13]; however, a variety of other more complex coupled food-web modules are possible [11]. In my previous work, therefore, I investigated how the development of ASS varies with the food-web structure at higher trophic levels (e.g., food-chain lengths and trophic levels in the juvenile and adult habitats) [11]. In the present study, I shift my focus to structural diversity at lower trophic levels. In particular, I focus here on multiple resource use. Multiple resource use is an important factor for community structure and dynamics, because it diversifies the trophic pathways and largely determines the amount of energy within food-webs [18], [19]. As a consequence, it is expected that multiple resource use would have significant impacts on ASS generated by an ontogenetic niche shift.

**Figure 1:**
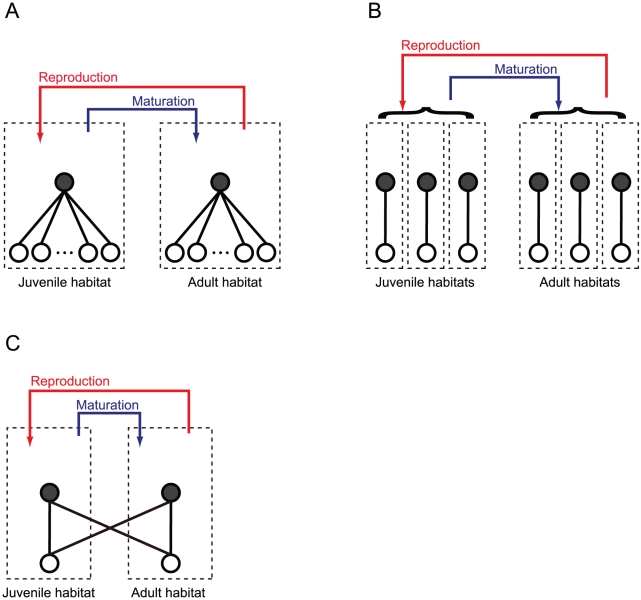
The multiple resource use in systems coupled by an ontogenetic niche shift. **(A) additional resources, (B) multiple habitats, and (C) interstage resource sharing.** In each panel, the juvenile and adult habitats are arranged on the left and right sides, respectively. The red and blue arrows represent reproduction and maturation flows, respectively. The solid circles represent the juveniles or adults, and the open circles represent their resources. The solid lines indicate trophic interactions with the resources.

With the term “multiple resource use,” in this study I define that juveniles and/or adults use more than two resources at each stage. The resource use may be of different types. In the present study, I consider the following three independent scenarios for model development. In the first scenario, I assume that juveniles and adults have “additional resources” (Fig. 1A) as a typical representative of alternative resource use [10], [20], [21]. In the second scenario, I assume that juveniles and adults have “multiple habitats” to colonize (Fig. 1B), that is, resource use in distinct habitats. This scenario can be applied to animals that do not always leave for the parental or natal habitats at an ontogenetic niche shift. Finally, I consider the scenario “interstage resource sharing” (Fig. 1C), assuming that the juveniles and adults can utilize the major resource for the other stage. In this scenario, the juvenile and adult habitats are not necessarily separated in space, but their food preference is stage-specific. Note that the aim of this study is not to analyze the model behaviors (e.g., population stability and species composition) in detail in each scenario. Instead, I aim to present analytical conditions for ASS resulting from an ontogenetic niche shift in the presence of multiple resource use.

## Methods

Throughout the modeling, I follow the previous studies [11], [12]. I assume that the resources exhibit logistic growth, all trophic interactions are linear, and both maturation and reproduction rates are proportional to food intake. The model extensions or modifications have been discussed in the literature by considering individual growth or nonlinearity [11]-[13] (also see Discussion), which are not accounted for in this study. Note also that when exploring the first model, I provide definitions of most parameters and analytical methods used in subsequent models.

## Results

### Scenario 1: Additional resources

First, I explore the model in which the juveniles and adults have additional resources (Fig. 1A). This model is described as follows:
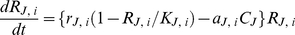
(1a)

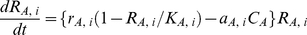
(1b)

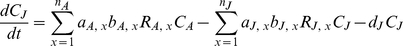
(1c)

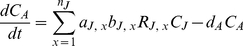
(1d)
*R_h_*_,*i*_ (*h* = *J* or *A*) is the resource abundance in a juvenile or adult habitat (*i* = 1, 2, …, *n_h_*), and *n_h_* is the resource number. *r_h_*_,*i*_ and *K_h_*_,*i*_ are the intrinsic growth rate and carrying capacity, respectively of the *i*th resource. *C_h_* is the juvenile or adult abundance. *a_h_*_,*i*_ and *b_h_*_,*i*_ are the consumption rate and energy conversion efficiency of the *i*th resource by the juveniles or adults, respectively. *d_h_* is the stage-specific death rate.

I examine the multiplicity of coexistence equilibria by performing zero-net-growth isocline (ZNGI) analysis. Consider the equilibrium state in which the juvenile and adult animals coexist with all resources. Using equations 1a and 1b, I obtain *R_J_*_,i_^*^ = *K_J_*_,*i*_(1−*a_J_*_,*i*_ *C_J_* ^*^/*r_J_*_,*i*_) and *R_A_*_,i_^*^ = *K_A_*_,*i*_(1−*a_A_*_,*i*_ *C_A_* ^*^/*r_A_*_,*i*_), respectively (asterisks denote equilibrium quantities). Substituting these expressions into *dC_A_*/*dt* = 0 and *d*(*C_J_*+*C_A_*)/*dt* = 0 yields two ZNGIs,
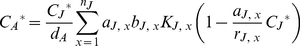
(2a)

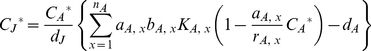
(2b)Hereafter, I denote the equations 2a and 2b as ZNGI_A_ and ZNGI_J_, respectively. The intersections of the two ZNGIs determine the coexistence equilibria. One solution is always trivial; for this solution, *C_J_* ^*^ = *C_A_* ^*^ = 0. The point in the analysis is that *C_J_* ^*^ and *C_A_* ^*^ are expressed as upward-convex quadratic functions of each other. Therefore, at most three coexistence equilibria are observed when ASS exist: one is an unstable equilibrium and the other two are stable ones (stable equilibrium point or stable periodic orbits; [11]-[13]).

The coexistence equilibria are obtained by solving a cubic equation *F*(*C_J_* ^*^) = *Λ*_1_(*C_J_* ^*^)^3^+*Λ*_2_(*C_J_* ^*^)^2^+*Λ*_3_ *C_J_* ^*^+*Λ*_4_ = 0, which is derived by substituting ZNGI_A_ in ZNGI_J_ (note that one solution is trivial). A necessary condition for the existence of ASS is that this equation has three positive solutions. Using the discriminant of a cubic equation, this condition is given as
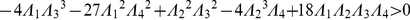
(3a)The following conditions are also imposed to ensure that the values of equilibrium abundance are positive:
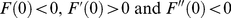
(3b)when *Λ*_1_>0. Parameter space for ASS can be numerically evaluated by using inequalities 3a and 3b.

**Figure 2:**
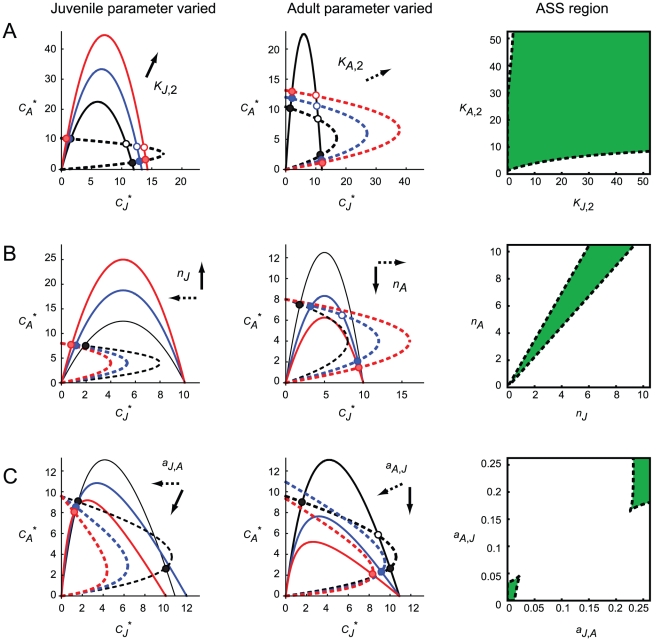
Parameter-dependence of the zero-net-growth isoclines (ZNGIs) and alternative stable states (ASS). (A) additional resources, (B) multiple habitats, and (C) interstage resource sharing. In each scenario, the left and central columns show the results of ZNGI analysis. The solid and dotted lines represent ZNGI_A_ and ZNGI_J_, respectively. The black lines are for the default parameter settings as described below. The blue and red lines represent ZNGI when one juvenile- or adult-specific parameter is increased. The solid and dotted arrows roughly denote the shift direction of ZNGI_A_ and ZNGI_J_, respectively, with an increase in the corresponding parameter. The solid and open circles represent stable and unstable equilibria, respectively. The blue and red circles indicate the intersections on a ZNGI of the same color. The right panel shows the analytical results for ASS in a corresponding two-parameter space. ASS exist in the green region. (A) *n_h_* = 2; left: *K_J_*_,2_ = 10, 20, or 30 and *K_A_*_,2_ = 10; center: *K_J_*_,2_ = 10 and *K_A_*_,2_ = 10, 20, or 30; right: *K_J_*_,2_ and *K_A_*_,2_ are variables. (B) *K_h_* = 10; left: *n_J_* = 2, 3, or 4 and *n_A_* = 2; center: *n_J_* = 2 and *n_A_* = 2, 3, or 4; right: *n_J_* and *n_A_* are variables. (C) *K_h_* = 15; left: *a_J_*_,*A*_ = 0.01, 0.05, or 0.1 and *a_A_*_,*J*_ = 0.01; center: *a_J_*_,*A*_ = 0.01 and *a_A_*_,*J*_ = 0.01, 0.05, or 0.1; right: *a_J_*_,*A*_ and *a_A_*_,*J*_ are variables. The other parameter values are set *r_h_* (or *r_h_*_,*i*_) = 1, *a_h_* (or *a_h_*_,*i*_) = 0.1, *b_h_* (or *b_h_*_,*i*_) = 0.5, and *d_h_* (or *d_h_*_,*i*_) = 0.1.

Here, I briefly show the parameter-dependence of the occurrence of ASS. For presentation, I simply assume that the juveniles and adults have two resources (i.e., *n_h_* = 2) and vary the productivity of the second resource *K_J_*_,2_ or *K_A_*_,2_ while keeping the other parameters fixed. The ZNGI analysis shows that ZNGI_A_ (or ZNGI_J_) shifts to the upper right with an increase in *K_J_*_,2_ (or *K_A_*_,2_) in the space of *C_J_* ^*^ and *C_A_* ^*^ (left or center panel in Fig. 2A), as illustrated by ∂*C_A_* ^*^/∂*K_J_*_,2_>0 (or *∂C_J_* ^*^/*∂K_A_*_,2_>0). These behaviors of the ZNGIs indicate that ASS exist when both *K_J_*_,2_ and *K_A_*_,2_ are sufficiently large but not when they differ considerably. This is illustrated by the analytical approach using inequalities 3a and 3b (right panel in Fig. 2A). Thus, it is suggested that, all other things being equal, the relative balance of the total resource availability in the juvenile and adult habitats is essential for the development of ASS. The qualitative results were basically the same when the juveniles or adults use more than two resources (not shown) or even when the additional resources are allochthonous subsidies (Supporting Information S1).

### Scenario 2: Multiple habitats

Next, I consider the situation where the juveniles and adults can colonize several habitats, where they exploit one resource (Fig. 1B). I assume here that colonization is a random process. The model is described as follows.
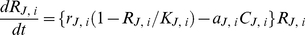
(4a)

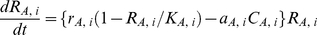
(4b)

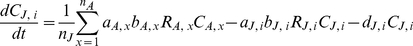
(4c)

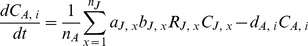
(4d)
*i* denotes the *i*th habitat, and *n_h_* (*h* = *J* or *A*) is the stage-specific habitat (or resource) number. For analytical tractability, I also assume that parameter values are identical in all juvenile or adult habitats (i.e., *r_h_*_,*i*_ = *r_h_*, *K_h_*_,*i*_ = *K_h_*, *a_h_*_,*i*_ = *a_h_*, *b_h_*_,*i*_ = *b_h_* and *d_h_*_,*i*_ = *d_h_*). Under these conditions, *R_h_*_,i_^*^ = *R_h_* ^*^ and *C_h_*_,i_^*^ = *C_h_* ^*^.

From equations 4a and 4b, I obtain *R_J_*_,i_^*^ = *K_J_*_,*i*_(1−*a_J_*_,*i*_ *C_J_* ^*^/*r_J_*_,*i*_) and *R_A_*_,i_^*^ = *K_A_*_,*i*_(1−*a_A_*_,*i*_ *C_A_* ^*^/*r_A_*_,*i*_), respectively. Substituting these expressions into *dC_A_*/*dt* = 0 and *d*(*C_J_*+*C_A_*)/*dt* = 0 yields the following two ZNGIs,
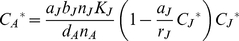
(5a)

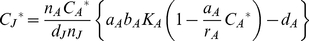
(5b)Here, I focus on the effect of varying *n_J_* or *n_A_* on the existence of ASS. The ZNGI analysis shows that changes in *n_J_* or *n_A_* affects both ZNGI_A_ and ZNGI_J_: ZNGI_A_ and ZNGI_J_ shift upward and to the left, respectively, with an increase in *n_J_* in the space of *C_J_* ^*^ and *C_A_* ^*^ (left panel in Fig. 2B), while they shift downward and to the right with an increase in *n_A_* (center panel in Fig. 2B). This is because an increase in the juvenile (or adult) habitat number not only increases (or dilutes) the maturation flow to an adult habitat but also dilutes (or increases) the reproduction flow (i.e., juvenile recruitment) to a juvenile habitat. Thus, changes in the stage-specific habitat numbers may influence the relative balance of the resource availability in the juvenile and adult habitats (thus, the development of ASS) more significantly than do local environmental changes (Fig. 2A). This is supporting by the mathematical analysis showing that ASS may occur in the relatively limited parameter region where *n_J_* and *n_A_* are comparable (right panel in Fig. 2B; note that *n_h_* is a noninteger in this analysis).

This model should be extended to include the spatial heterogeneities in stage-specific local environmental conditions. Here, I only briefly present the preliminary numerical results. For simplicity, I assume that both the juveniles and adults have two habitats, and introduce environmental heterogeneity as a difference in productivity between the two juvenile habitats. The results indicate that the system has at least three ASS for some parameter settings (Supporting Information S2): one is an adult-dominated state, and the other two are juvenile-dominated states in which either of the two juvenile subpopulations dominates the other depending on the initial conditions. Therefore, it is expected that there exist numerous stable states of spatial community structure when juveniles and adults can colonize multiple habitats with different environmental conditions. For a better understanding of this phenomenon, further detailed analyses are necessary; these will be conducted in future work.

### Scenario 3: Interstage resource sharing

Finally, I assume that the juveniles and adults can utilize the major resources of the other life-history stage (Fig. 1C). The model is described as follows:
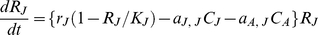
(6a)

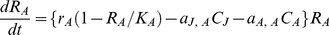
(6b)

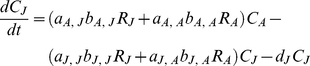
(6c)

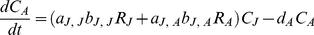
(6d)
*a_h_*_,*i*_ (*h*, *i* = *J* or *A*) is the rate of consumption of resource *R_i_* by the juveniles or adults. *b_h_*_,*i*_ is the conversion efficiency for *a_h_*_,*i*_.

From equations 6a and 6b, I obtain *R_J_*_,i_^*^ = *K_J_*_,*i*_(1−*a_J_*_,*i*_ *C_J_* ^*^/*r_J_*_,*i*_) and *R_A_*_,i_^*^ = *K_A_*_,*i*_(1−*a_A_*_,*i*_ *C_A_* ^*^/*r_A_*_,*i*_), respectively. Substituting these expressions into *dC_A_*/*dt* = 0 and *d*(*C_J_*+*C_A_*)/*dt* = 0 yields the following two ZNGIs,
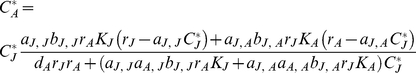
(7a)

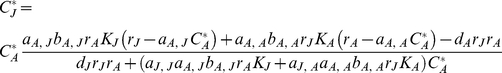
(7b)ZNGI_A_ and ZNGI_J_ are fractional functions of each other; their numerator and denominator are upward-convex quadratic and linearly increasing functions, respectively. Here, I focus on the effect of varying *a_J_*_,*A*_ or *a_A_*_,*J*_ on the development of ASS. In the space of *C_J_* ^*^ and *C_A_* ^*^, ZNGI_A_ and ZNGI_J_ generally shifts to the lower left and downward with an increase in *a_J_*_,*A*_ or *a_A_*_,*J*_, respectively (left and center panels in Fig. 2C). Because of the complexity of the functions, however, it is difficult to fully understand the development of ASS by using ZNGI analysis. However, the mathematical analysis is feasible because the set of ZNGI_A_ and ZNGI_J_ can be represented by a cubic equation (not shown). The analytical results illustrate that ASS are generally suppressed when either *a_J_*_,*A*_ or *a_A_*_,*J*_ or both are large (right panel in Fig. 2C). The mechanism of the suppression of ASS can be explained as follows. Suppose the amount of the major juvenile resource increases. If the adults exploit the juvenile resource with high efficiency, the reproduction flow is enhanced, and thus, the juvenile resource is affected negatively. This negative feedback interrupts the positive one necessary for ASS. ASS redevelop when both *a_J_*_,*A*_ and *a_A_*_,*J*_ are very large (right panel in Fig. 2C). This is simply because the juveniles and adults exchange their major resources.

## Discussion

In this study, I theoretically investigated the development of ASS resulting from ontogenetic habitat coupling in the presence of multiple resource use in different scenarios (Fig. 1). All the results demonstrated that multiple resource use critically affects coupled food-web dynamics and the development of ASS (Fig. 2). Nakazawa [11] indicated that the food-web structure at higher trophic levels (e.g., food-chain length and the trophic positions of juveniles and adults) significantly influence the development of ASS by altering the strength of stage-specific top-down control [11]. Taken together, these results suggest that both bottom-up and top-down controls of life-history stages should be elucidated for a better understanding of the community structure and dynamics mediated by an ontogenetic niche shift.

Previous theoretical studies, in which one-consumer-two-resource systems were considered, suggest that the relative balance of the juvenile and adult habitat productivities is crucial for the development of ASS [12], [13]. This criterion is generalized in the present study. In the first model, I showed that the total resource availability (including all available autochthonous and allochthonous resources) of the juvenile and adult stages should be balanced for the existence of ASS (Figs. 2A and Supporting Information S1). Resource availability is determined not only by habitat productivity or subsidy input but also by consumer mobility because highly mobile animals have access to abundant resources in a large foraging area. In the multiple-habitat model, ASS exist when the juveniles and adults have a comparable number of habitats (Fig. 2B). These results suggest that the likelihood of ASS is high when the total resource availability is balanced between the juvenile and adult habitats at large spatial scales. These are natural but nontrivial extensions of the previous criterion and may provide an easy-to-use indicator of the likelihood of ASS in complex situations.

I also showed that interstage resource sharing generally suppresses ASS (Fig. 2C). This result suggests that the stage specificity of resource utilization is another good indicator of the likelihood of ASS. The stage specificity of resource utilization may be determined not only by stage-specific resource preference but also by stage-dependent foraging mobility. For example, adults (with possibly high mobility) have the ability to search for resources in a large area, while juveniles may exploit the resources within a localized area. In this case, the juveniles have to share the resource with the adults (but not vice versa), and thus, the likelihood of ASS will decrease with an increase in the rate of juvenile resource exploitation by the adults. Interstage resource sharing may also occur when an ontogenetic niche shift occurs as a result of a gradual individual growth. For example, some fish change from planktivory to benthivory as they grow. In this case, adults may exploit both plankton and benthos, while juveniles may not use benthos [22]. De Roos et al. [23] investigated such situations by using a physiologically structured model and observed ASS; however, the underlying mechanism could not be fully elucidated because of the model complexity. On the other hand, because my model is a simplified conceptual representation of a gradual ontogenetic niche shift, and thus helps understand the basic mechanisms underlying the development and inhibition of ASS in such situations.

My models were developed for the specific purpose of analytically identifying the conditions for ASS in the presence of multiple resource use. I therefore purposely formulated simple analytical models without incorporating additional factors affecting population dynamics. To better understand the occurrence of ASS in reality, therefore, the models need to be modified or extended as in the following examples. First, one may be concerned with the case in which trophic interactions are nonlinear. If the functional forms are nonlinear due to a long handling time or strong interference competition, it decreases the likelihood of ASS (see [11], [12]). This is because maturation or reproduction becomes less food-dependent, thereby suppressing the positive feedback necessary for ASS. If the relationship between food intake and maturation or reproduction is nonlinear, it may also suppress the development of ASS by the same mechanism. Second, adaptive behaviors may also be considered especially when multiple resources are available. Takimoto [24] theoretically demonstrated that an adaptive ontogenetic niche shift (i.e., juveniles delay or advance the niche shift timing depending on juvenile resource availability) has a stabilizing effect (i.e., negative feedback regulation). His results imply that an adaptive ontogenetic niche shift may suppress ASS, because it has negative feedback regulation. Meanwhile, foraging adaptation within stages will generally enhance stage-specific resource exploitation, and thus, increase or decrease the likelihood of ASS by changing the relative balance between the resource availability for juveniles and adults. Third, real life histories of animals undergoing an ontogenetic niche shift are much more complicated than I assumed here. In particular, individual growth is crucial for maturation and reproduction. Guill [13] showed that ASS can occur even if the juveniles have individual growth. Therefore, I expect that my predictions would be generally robust (see also [11] for further discussion). These model limitations and extensions, including those not mentioned here, can be overcome by performing computer simulations of more complex systems; although this is required for understanding ASS in specific real systems, it is beyond the scope of this study.

Finally, I like to emphasize that ontogenetic habitat coupling may produce more diverse and complex community dynamics in the presence of multiple resource use. It is known that a consumer of multiple resources may drive some of the resources extinct, or to very low levels, via apparent competition [25]. This may occur in my scenarios via apparent competition both within and across stages. In this study, I focused on examining parameter conditions for the occurrence of ASS in the specific structures of systems, without considering species compositional changes, by using the ZNGI analysis. Different analyses are therefore required for more completely understanding the detail behaviors of the proposed systems.

In conclusion, the present study demonstrated that the development of ASS is critically affected by the food-web structure at lower trophic levels; the development is determined by various factors such as spatial resource distribution, colonization area, consumer mobility, and stage-specificity of resource utilization. Currently, there are increasing concerns about anthropogenic impacts of eutrophication [26], habitat modifications (e.g., destruction and fragmentation; [27]), and interaction modificationss (e.g., changes in ontogeny and foraging behavior; [28]). It is hypothesized that the anthropogenic impact on animals undergoing an ontogenetic niche shift propagates spatially and may cause or prevent regime shifts in the regional community structure. My results will contribute to gaining a better understanding of the ecosystem resilience mediated by ontogenetic food-web coupling and provide useful insights into ecosystem management on large spatial scales.

## Supporting Information

Supporting Information S1 (0.11 MB DOC)

Supporting Information S2 (0.11 MB DOC)
