# Supplementary material for: Alternative Stable States Generated by Ontogenetic Niche Shift in the Presence of Multiple Resource Use
Source: PLoS One. 2011 Feb 8;6(2):e14667. doi: 10.1371/journal.pone.0014667 (PMC3035614; doi:10.1371/journal.pone.0014667)
Supplement: Supporting Information S1 — (0.11 MB DOC) [file pone.0014667.s001.doc]

**Supporting Information S1** Alternative stable states in the presence of allochthonous subsidies

In this appendix, I show that ASS can occur even when juveniles and adults exploit allochthonous subsidies as additional resources. Here, I simply assume that one autotroph and one allochthonous subsidy are available at each stage. The model is described as follows.

$\frac{dR_{J}}{dt}=\left\{r_{J}\left(1-R_{J}/K_{J}\right)-a_{J}C_{J}\right\}R_{J}$ (S1a)

$\frac{dR_{A}}{dt}=\left\{r_{A}\left(1-R_{A}/K_{A}\right)-a_{A}C_{A}\right\}R_{A}$ (S1b)

$\frac{dS_{J}}{dt}=i_{J}-e_{J}S_{J}-a'_{J}S_{J}C_{J}$ (S1c)

$\frac{dS_{A}}{dt}=i_{A}-e_{A}S_{A}-a'_{A}S_{A}C_{A}$ (S1d)

$\frac{dC_{J}}{dt}=\left(a_{A}b_{A}R_{A}+b'_{A}a'_{A}S_{A}\right)C_{A}-\left(a_{J}b_{J}R_{J}+b'_{J}a'_{J}S_{J}\right)C_{J}-d_{J}C_{J}$ (S1e)

$\frac{dC_{A}}{dt}=\left(a_{J}b_{J}R_{J}+b'_{J}a'_{J}S_{J}\right)C_{J}-d_{A}C_{A}$ (S1f)

*S_h_* (*h* = *J* or *A*) is the abundance of allochthonous subsidy into a juvenile or adult habitat. *i_h_* and *e_h_* are the supply and decay rates, respectively, of the subsidy. *a*’*_h_* and *b*’*_h_* are the consumption rate and conversion efficiency, respectively, of the subsidy by the juveniles or adults.

At the coexistence equilibrium, I obtain *R_h_*,^*^ = *K_h_*(1 - *a_h_C_h_*^*^/*r_h_*) and *S_h_*^*^ = *i_h_*/(*e_h_* + *a*’*_h_C_h_*^*^). Substituting these expressions into *dC_A_*/*dt* = 0 and *d*(*C_J_* + *C_A_*)/*dt* = 0 yields the following two ZNGIs,

$C_{A}^{*}=\frac{C_{J}^{*}}{d_{A}}\left\{a_{J}b_{J}K_{J}\left(1-\frac{a_{J}}{r_{J}}C_{J}^{*}\right)+\frac{b'_{J}a'_{J}i_{J}}{e_{J}+a'_{J}C_{J}^{*}}\right\}$ (S2a)

$C_{J}^{*}=\frac{C_{A}^{*}}{d_{J}}\left\{a_{A}b_{A}K_{A}\left(1-\frac{a_{A}}{r_{A}}C_{A}^{*}\right)-d_{A}+\frac{b'_{A}a'_{A}i_{A}}{e_{A}+a'_{A}C_{A}^{*}}\right\}$ (S2b)

ZNGI_A_ and ZNGI_J_ are expressed as complex functions of each others. However, the functions well approximate upward-convex quadratic functions for *C_J_*^*^ > 0 or *C_A_*^*^ > 0. Therefore, at most three coexistence equilibria are observed when ASS exist. I examine the effects of varying *i_J_* or *i_A_* on the development of ASS. The ZNGI analysis generally shows that ZNGI_A_ (or ZNGI_J_) shifts to the upper right with an increase in *i_J_* (or *i_A_*) in the space of *C_J_*^*^ and *C_A_*^*^ (left or center panel in Fig. S1), as illustrated by *∂C_A_*^*^/*∂i_J_* > 0 (or *∂C_J_*^*^/*∂i_A_* > 0). These behaviors of the ZNGIs indicate that ASS exist when *i_J_* and *i_A_* are both large and comparable in magnitude. We also confirmed this result by numerical simulations (right panel in Fig. S1). These results are qualitatively similar to those obtained in the model of additional autotrophic resources (Fig. 2A). In the simulations, the parameters are *r_h_* = 1, *K_h_* = 10, *a_h_* (or *a*’*_h_*) = 0.1, *b_h_* (or *b*’*_h_*) = 0.5, and *d_h_* = 0.1, and *e_h_* = 0.1. (left) *i_J_* = 5, 10, or 20 and *i_A_* = 5, (center) *i_J_* = 5 and *i_A_* = 5, 10, or 20, and (right) *i_J_* and *i_A_* are variables. Other notations are identical to those of Figure 2.

**Figure S1**
