# Supplementary material for: Alternative Stable States Generated by Ontogenetic Niche Shift in the Presence of Multiple Resource Use
Source: PLoS One. 2011 Feb 8;6(2):e14667. doi: 10.1371/journal.pone.0014667 (PMC3035614; doi:10.1371/journal.pone.0014667)
Supplement: Supporting Information S2 — (0.11 MB DOC) [file pone.0014667.s002.doc]

**Supporting Information S1** Alternative stable states in the multiple-habitat scenario with spatial environmental heterogeneity

In this appendix, I numerically show that more than two ASS can exist in the multiple-habitat scenario with spatial environmental heterogeneity. For simplicity, I assume that the juveniles and adults have two habitats (i.e., *n_h_* = 2) and that the productivities in the two juvenile habitats *K_J_*_,1_ and *K_J_*_,2_ are different. Under the condition, at least three ASS are found for some parameter settings (Fig. S1). One is an adult-dominated state, where the system converges to a stable point attractor (State 1). The others are juvenile-dominated states, where the system exhibits stable periodic orbits dominated by either of the two juvenile subpopulations (States 2 and 3). In the simulations, the parameters are *K_J_*_,1_ = *K_A_*_,1_ = *K_A_*_,2_ = 10 and *K_J_*_,2_ = 30. Other conditions are identical to those corresponding to Figure 2B. The thick and broken (or dotted) lines represent the total and subpopulation abundances of the juveniles or adults, respectively.

**Figure S1**
